# Supplementary material for: Biological Characteristics of Verticillium dahliae MAT1-1 and MAT1-2 Strains
Source: Int J Mol Sci. 2021 Jul 1;22(13):7148. doi: 10.3390/ijms22137148 (PMC8269371; doi:10.3390/ijms22137148)
Supplement: Supplementary file 1 [file ijms-22-07148-s001.zip › Additional Information.pdf]

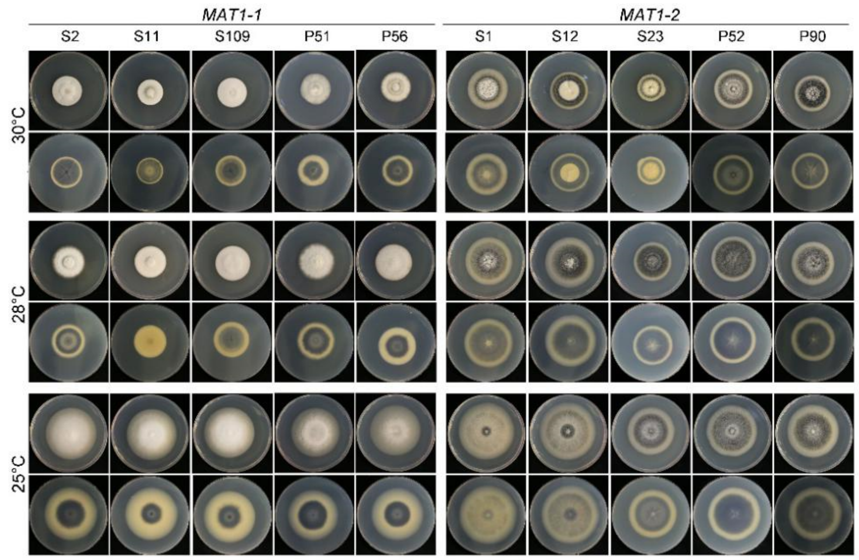

Figure S1 Growth phenotype of *MAT1-1* and *MAT1-2* strain populations at 25 °C, 28 °C, and 30 °C on PDA medium after culturing for 15 days. P and S indicate strains isolated from potato and sunflower, respectively.

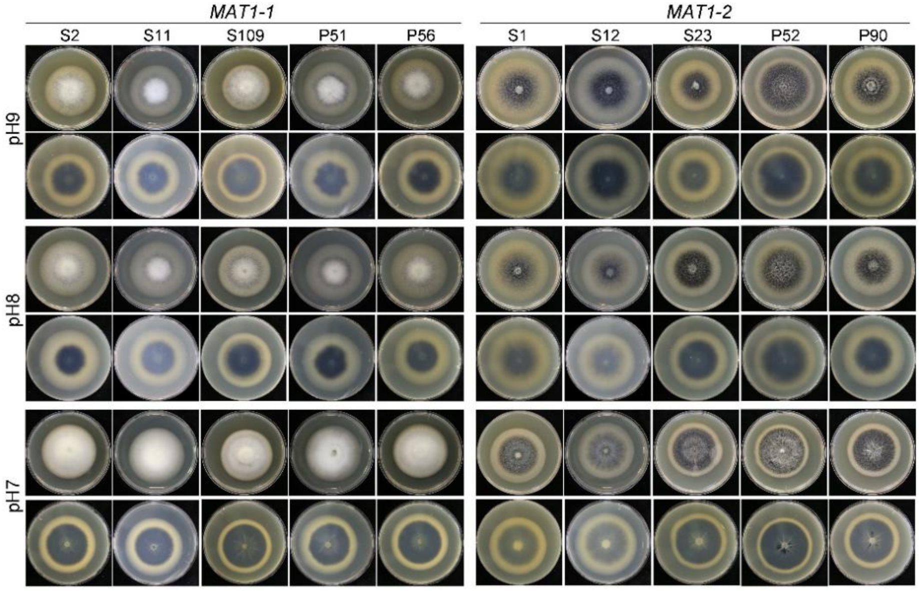

Figure S2 Growth phenotype of *MAT1-1* and *MAT1-2* strain populations on PDA after culturing for 15 days in medium adjusted to pH 7, pH 8, and pH 9. P and S indicate strains isolated from potato and sunflower, respectively.

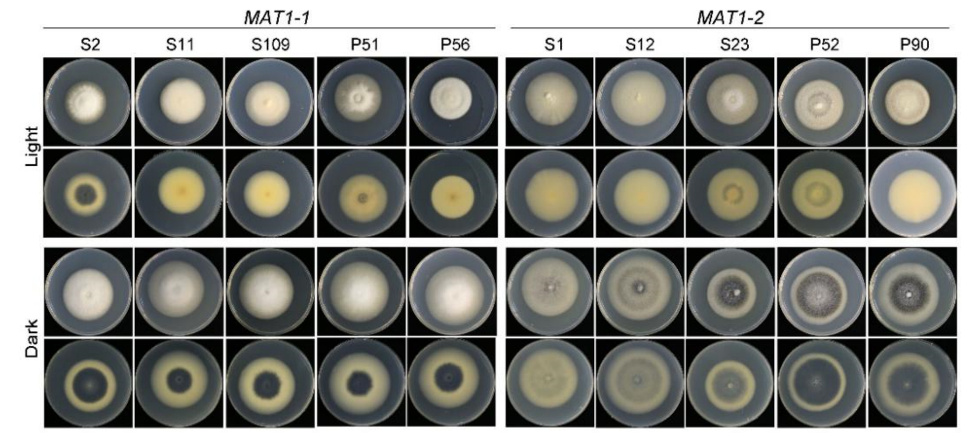

Figure S3 Growth phenotype of *MAT1-1* and *MAT1-2* strain populations under light and dark conditions for 15 days. P and S indicate strains isolated from potato and sunflower, respectively.

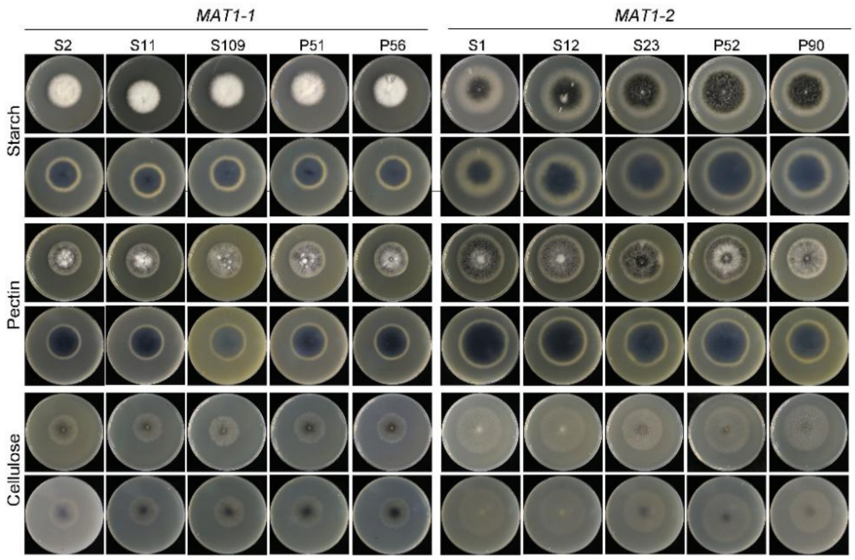

Figure S4 Growth phenotype of *MAT1-1* and *MAT1-2* strain populations after culturing for 15 days in media types containing different carbon sources. P and S indicate strains isolated from potato and sunflower, respectively.

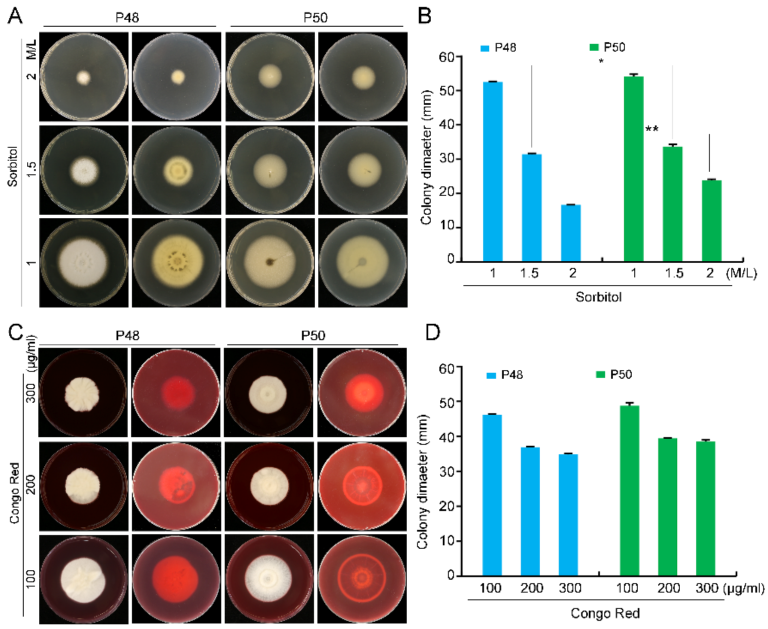

Figure S5 Growth of strains P48 and P50 under two stress tolerance conditions. (**A**) and (**B**) Growth phenotype and colony diameter of P48 and P50 in sorbitol medium after culturing for 15 days. **(C) and (D)** Colony diameter of P48 and P50 in response to cell wall stress (Congo red) after culturing for 15 days. Asterisks * and ** indicate significant differences $P < 0.05$ and $P < 0.01$, respectively, according to unpaired Student's *t*-test.

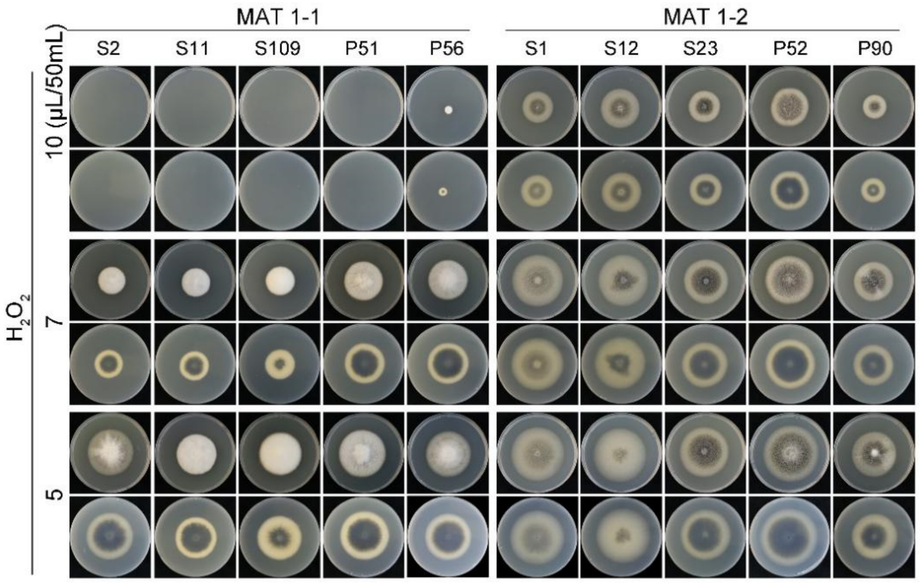

Figure S6 Growth of *MAT1-1* and *MAT1-2* strain populations under conditions of oxidative stress. *MAT1-1* and *MAT1-2* strain populations isolated from sunflower

(S) and potato (P) were cultured on medium with different concentrations of H_2O_2 (0.9, 1.2, 1.8 mmol/L) for 15 days.
